# Aspergillusidone G Potentiates the Anti-Inflammatory Effects of Polaprezinc in LPS-Induced BV2 Microglia: A Bioinformatics and Experimental Study

**DOI:** 10.3390/md22070324

**Published:** 2024-07-19

**Authors:** Fangfang Ban, Longjian Zhou, Zhiyou Yang, Yayue Liu, Yi Zhang

**Affiliations:** 1 Guangdong Provincial Key Laboratory of Aquatic Product Processing and Safety, Guangdong Provincial Engineering Laboratory for Marine Biological Products, Guangdong Provincial Center for Modern Agricultural Scientific Innovation, Shenzhen Institute of Guangdong Ocean University, Zhanjiang Municipal Key Laboratory of Marine Drugs and Nutrition for Brain Health, Research Institute for Marine Drugs and Nutrition, College of Food Science and Technology, Guangdong Ocean University, Zhanjiang 524088, China; banfangfang@126.com (F.B.); yang_zhiyou@sina.com (Z.Y.); yayue_liu@163.com (Y.L.); 2 Southern Marine Science and Engineering Guangdong Laboratory (Zhanjiang), Zhanjiang 524088, China; 3 Collaborative Innovation Center of Seafood Deep Processing, Dalian Polytechnic University, Dalian 116034, China

**Keywords:** neuroinflammation, polaprezinc, aspergillusidone G, synergistic strategy, NF-κB signaling pathway

## Abstract

Neuroinflammation is one of the main mechanisms involved in the progression of neurodegenerative diseases (NDs), and microglial activation is the main feature of neuroinflammation. Polaprezinc (Pol), a chelator of L-carnosine and zinc, is widely used as a clinical drug for gastric ulcers. However, its potential effects on NDs remain unexplored. In LPS-induced BV-2 microglia, we found that Pol reduced the generation of NO and ROS and revealed inhibited expression of iNOS, COX-2, and inflammatory factors such as IL-6, TNF-α, and 1L-1β by Pol using qRT-PCR and Western blotting. These effects were found to be associated with the suppression of the NF-κB signaling pathway. Moreover, we evaluated the potential synergistic effects of aspergillusidone G (Asp G) when combined with Pol. Remarkably, co-treatment with low doses of Asp G enhanced the NO inhibition by Pol from approximately 30% to 80% in LPS-induced BV2 microglia, indicating a synergistic anti-inflammatory effect. A bioinformatics analysis suggested that the synergistic mechanism of Asp G and Pol might be attributed to several targets, including NFκB1, NRF2, ABL1, TLR4, and PPARα. These findings highlight the anti-neuroinflammatory properties of Pol and its enhanced efficacy when combined with Asp G, proposing a novel therapeutic strategy for managing neuroinflammation in NDs.

## 1. Introduction

With the ongoing increases in life expectancy, the global socioeconomic impact of neurodegenerative diseases (NDs) such as Alzheimer’s disease, Parkinson’s disease, Amyotrophic lateral sclerosis, and Huntington’s disease is rising considerably. NDs share many common yet diverse pathological and clinical characteristics, including the selective susceptibility of brain regions and aggregation of different proteins. Neuroinflammation, however, is a commonly shared feature of NDs [1]. Neuroinflammation was previously thought to be the result of protein aggregations in the central nervous system (CNS). However, growing evidence indicates that immune signaling may not only be the result of protein aggregation in the CNS but may lead to the accumulation of aggregates at the early stages of the disease process [2,3,4]; in particular, growing immune-related genetic mutations have been suggested to be risk factors for neurodegeneration [5].

The activation of microglia is a primary source of neuroinflammation in the CNS. Microglia are the resident macrophages of the CNS, playing a crucial role not only in neurogenesis, neuronal plasticity, and regeneration but also as a first line of immune defense in any kind of brain injury [6,7,8]. Microglia are in a “resting” state under physiological conditions and present a ramified morphology that transforms into an amoeboid morphology in response to injury or inflammatory stimuli [9,10,11]. The amoeboid morphology reflects a highly activated state, which leads to the occurrence of chronic inflammation and the deterioration of diseases if not restored promptly. Reactive amoeboid microglia are characterized by rapid proliferation and the production of a wide spectrum of cytokines, including interleukin (IL)-6, IL-1β, and tumor necrosis factor (TNF-α); chemokines, including C-C motif chemokine ligand (CCL) 1, CCL5, and C-X-C motif chemokine ligand 1 (CXCL1); and other mediators, including prostaglandins, nitric oxide (NO), and reactive oxygen species (ROS) [8]. Various stimuli have been demonstrated to induce the activation of microglia both in vitro and in vivo, and the endotoxin hypothesis suggests that lipopolysaccharide (LPS), the main component of endotoxin, may be involved in neurodegenerative processes by promoting microglial activation and some other related pathologies [12,13,14]. LPS is a major component of the outer membrane of Gram-negative bacteria [15], which directly or indirectly activates microglia, damaging neurons via the actions of NO, oxidants, and cytokines and phagocytosis of synapses and neurons. Toll-like receptor 4 (TLR4), the ligand of LPS, triggers inflammatory pathways such as nuclear factor kappa-B (NF-κB) signaling [8]. In short, the prospect of targeting microglia for the treatment of NDs is tantalizing.

Based on this hypothesis that pathological protein aggregation is the main initiator of neurodegeneration, most immunotherapies for NDs have harnessed the immune system to clear pathological protein aggregates in the past few decades, and clinical trials have shown that most of these therapies appeared to reduce the aggregates but failed to stop the disease’s progression [16]. Due to the limited success of conventional therapeutic approaches, blocking or enhancing the inflammatory signaling pathways involved in neurodegeneration, including many receptors (i.e., TLR, P2Y purinoceptor 6 (P2Y6), colony-stimulating factor 1 receptor (CSF-1R)), signal transducers (i.e., Janus kinase (JAK), p38 mitogen-activated protein kinases (p38 MAPK), extracellular regulated protein kinases (ERK)), activators of transcription (i.e., signal transducer and activator of transcription (STAT), NF-κB), and inflammatory mediators (i.e., granulocyte-CSF (GM-CSF), TNF-α, NO) is considered a promising strategy for the treatment of NDs, many of which have shown promising results in animal models or clinical trials [16]. Given the disadvantages of new drug development for NDs, such as the high risks, long cycles, and high investment costs involved, the strategy of “the new use of old drugs” is considered a great option to accelerate the process of new drug discovery.

L-carnosine is a dipeptide alkaloid composed of β-alanine and L-histidine, mainly extracted from mammalian muscle and also found in high levels in some marine fish. L-carnosine is also found in the olfactory receptor neurons in the brain [17], and is hypothesized to be involved in the modulation of neurotransmission and maintenance of homeostasis mainly through the antioxidant, metal-chelating, and antiglycative properties in certain brain structures [18]. Zinc exists in structural or labile forms in the CNS, which is crucial to the control of the physiological and pathophysiological brain function [19,20]. The relationship between zinc levels in the brain and the risk of NDs is controversial. One view is that excess zinc is strongly associated with the development of NDs, as elevated intraneuronal zinc is critical for initiating necrosis, autophagy, and apoptosis [20]. Based on this view, in vitro and in vivo studies have shown that the neuroprotective mechanism of L-carnosine may be related to the regulation of chelation of excess labile zinc produced by the CNS under pathological conditions [21,22,23]. Interestingly, the chelation of L-carnosine with zinc results in the formation of a specific polymer, polaprezinc (Pol), which is currently widely used as a clinical drug for gastric ulcers and zinc supplementation. A very important reason why Pol can treat gastric ulcers is due to its stable presence in the stomach and its ability to adhere to ulcerous sites in a much better way than either zinc or L-carnosine alone [24]. However, its effects on NDs have not been reported. Both L-carnosine and zinc are associated with NDs, and Pol possesses anti-inflammatory and antioxidant properties, all of which prompt us to consider whether Pol is also applicable in the treatment of NDs. Another view is that zinc deficiency is associated with a high risk of NDs. This view stems from the analysis of several clinical data [25,26] and the beneficial effects of exogenous zinc through a variety of mechanisms such as oxidative stress, neuronal viability, α-synuclein, recombinant Parkinson’s disease protein 2 (PARK2), and synaptic plasticity under the influence of neurotrophic factors [27,28,29,30,31,32]. This also supports the investigation of Pol for ND treatment.

Peptides have shown considerable potential in synergistic combinations with small-molecule drugs, particularly in the context of enhanced therapeutic efficacy. This synergy is not limited to antimicrobial applications [33,34] but extends to various therapeutic areas, including NDs [35]. For example, the study by Chio et al. found the combination of the dipeptide nitrile CD24 and curcumin showed antimicrobial activity better than that of CD24 alone [34]. Another study found that the peptide TFP5, derived from the p35 protein, when co-administered with a Cdk5 inhibitor roscovitine, showed significant neuroprotective effects in models of Parkinson’s disease [35]. Peptides may contribute to their intermediate nature by extending “beyond size”, combining the advantages of both small-molecule drugs (e.g., better permeability) and therapeutic proteins (selectivity, target potency) and excluding their disadvantages, such as their adverse side effects, drug–drug interactions, and membrane impermeability, respectively [36]. This prompted us to consider whether the dipeptide complex Pol could also synergize well with other compounds as well. Thus, in this research, we tested the combination of Pol and Aspergillusidone G (Asp G). Asp G consists of two 2,4-dihydroxybenzoic acid rings linked together by an ester bond and is a novel natural product isolated from the marine fungus *Aspergillus unguis* DLEP2008001, with good biological activity [37].

In this study, we aimed to investigate the anti-neuroinflammatory activity of Pol and its potential synergistic interaction with Asp G in LPS-induced BV2 microglia, elucidate the underlying mechanisms, and evaluate the therapeutic potential of this combination for NDs.

## 2. Results

### 2.1. Pol Possessed Acceptable ADMET and Drug-likeness Properties in General

**Figure 1:**
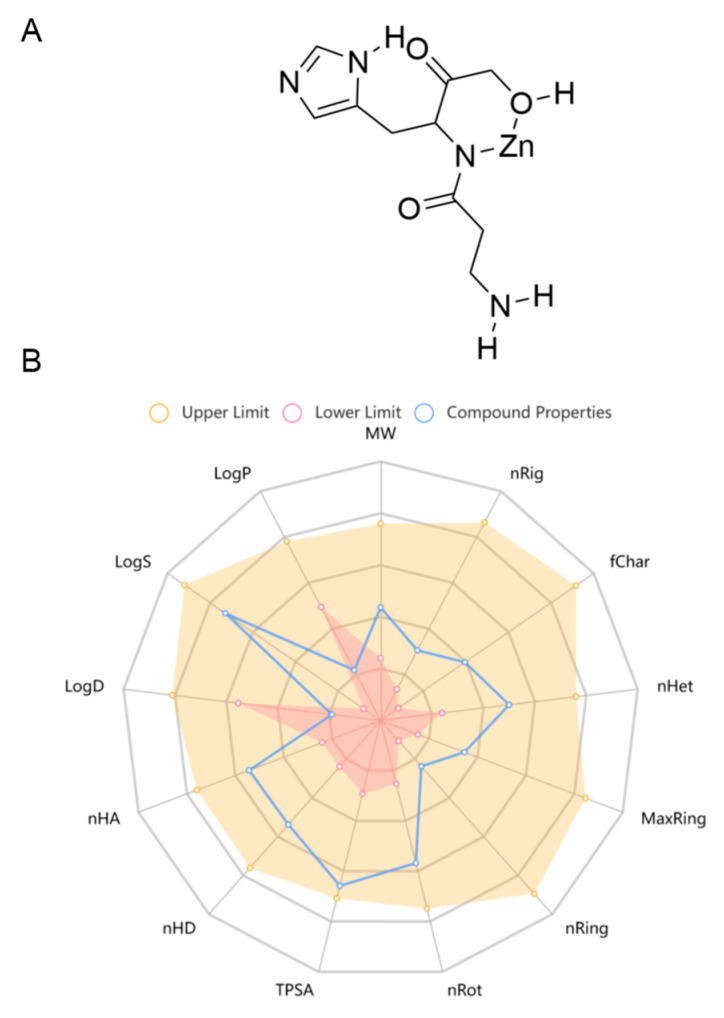
The chemical structure (**A**) and physicochemical properties (**B**) of Pol through ADMETlab 2.0.

Pol was recently approved for the first time in Japan, which has been widely used to treat gastric ulcers. In China, it is used as a prescription drug in clinical practice for gastric ulcers. Pol has a high safety profile, with no significant adverse effects seen at therapeutic doses of up to 300 mg daily, and its recommended oral dosage is 75 mg each time, twice a day for adults [38]. As there is a lack of pharmacokinetic studies on Pol, the online tool ADMETlab 2.0 (https://admetmesh.scbdd.com/service/evaluation/cal), accessed on 30 May 2024, was used to assist in the prediction of its ADMET properties, including its absorption, distribution, metabolism, excretion, and toxicity. The results showed that Pol possessed acceptable physicochemical, ADMET, and drug-likeness properties in general (Figure 1, Table 1 and Table S1). For example, it displayed excellent performance in blood–brain barrier (BBB) penetration. Meanwhile, it had acceptable safety profiles, generally performing well on most metrics (i.e., hERG blockers, human hepatotoxicity, drug-induced liver injury, AMES toxicity, rat oral acute toxicity, maximum recommended daily dose, carcinogenicity, eye corrosion, eye irritation, and respiratory toxicity). Additionally, it also presented excellent performance in its drug-likeness properties, including the Lipinski rule, the Pfizer rule, the GSK rule, and the golden triangle. Unfortunately, Pol showed some disadvantages, such as poor caco-2 permeability and PGP–substrate, CL, and skin sensitization. Overall, Pol has the potential to develop into a brain drug.

### 2.2. Pol Alleviated NO Burst in LPS-Induced BV2 Microglia

**Figure 2:**
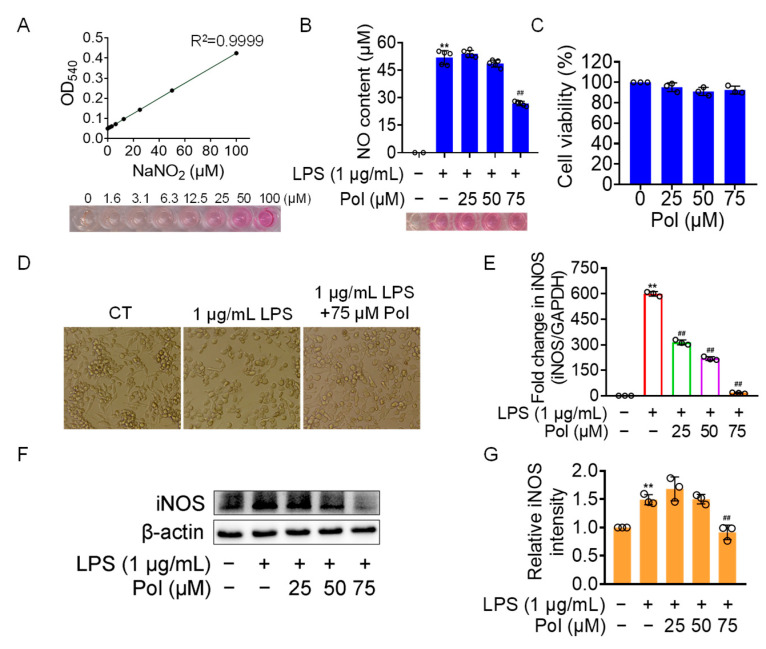
Pol relieved the generation of NO in LPS-induced BV2 microglia. BV2 microglia were exposed to different concentrations of Asp G for 1 h prior to the treatment of 1 μg/mL of LPS for another 12 h (**E**) or 24 h (**B**,**D**,**F**,**G**). (**A**) The standard curve of the NO content with NaNO_2_ as the standard was detected by the Griess reagent. (**B**) No content of BV2 microglia treated with Pol and LPS was determined by the Griess reagent. (**C**) Cell viability of BV2 microglia after exposure to Pol for 24 h was tested using CCK8. (**D**) Cellular morphology of BV2 microglia exposed to Pol and LPS was observed under a microscope. (**E**) The transcriptional expression of the iNOS gene in BV2 microglia exposed to Pol and LPS was detected using qRT-PCR. (**F**) The protein expression level of iNOS in BV2 microglia treated with Pol and LPS was tested using Western blotting. (**G**) Statistics graph of the protein expression of iNOS. Results are presented as means ± SD (*n* = 2 independent experiments for (**A**), *n* = 5 independent experiments for (**B**,**D**), *n* = 3 independent experiments for (**C**,**E**–**G**); ** *p* < 0.01 indicates a statistically significant difference compared to the untreated control group and ^##^ *p* < 0.01 indicates a statistically significant difference compared to the LPS group. The results were statistically analyzed using a one-way ANOVA.

NO has been implicated in modulating a multitude of physiological functions such as synaptic plasticity, vasoprotective effects, and cytostatic effects on undesired microbes, parasites, or tumor cells [39]. However, abnormal NO production is likely to contribute to the occurrence of related diseases. Excessive NO generation by activated microglia in the CNS is specifically associated with neuroinflammatory and neurodegenerative conditions. Therefore, it is a desirable therapeutic strategy to manage various neuroinflammatory disorders by controlling excessive NO production. Here, we aimed to investigate the effectiveness of Pol on neuroinflammation by employing LPS-induced BV2 microglia as a neuroinflammatory model. As the results showed, 75 μM of Pol attenuated above 50% of the NO generation in LPS-exposed BV2 microglia (Figure 2A,B) and significantly blocked its amoeboid activation, which is a morphology involving the enlargement of cell bodies and the shortening of cell processes (Figure 2D). Meanwhile, Pol did not exhibit a cytotoxic effect, even at the highest concentration (75 μM of Pol) (Figure 2C). Further, Pol also showed an inhibitory effect on the LPS-induced gene (Figure 2E) and protein (Figure 2F,G) and high expression levels of inducible nitric oxide synthase (iNOS), which mediated the synthesis of NO. In short, these data suggest that Pol reduced the production of NO, possibly by restraining the transition of BV2 microglia from the resting state to active state.

### 2.3. Pol Relieved the ROS Generation and Expression of Proinflammatory Cytokines and COX-2

**Figure 3:**
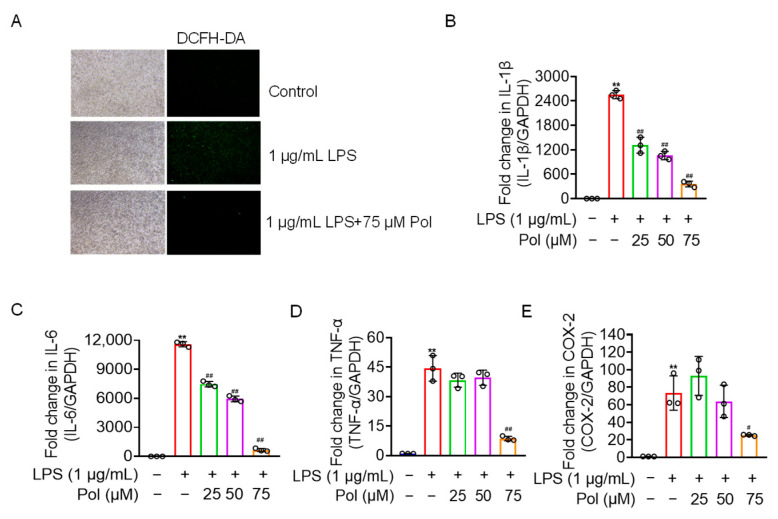
Pol relieved ROS, the expression of proinflammatory cytokines, and COX-2. BV2 microglia were exposed to the indicated concentration of Pol for 1 h and then treated with 1 μg/mL of LPS for another 12 h (**B**–**E**) or 24 h (**A**). (**A**) ROS fluorescence of BV2 microglia after the treatment with Pol and LPS was measured using a DCFH-DA probe with a fluorescence microscope. Transcriptional levels of proinflammatory factors in BV2 microglia exposed to Pol and LPS were measured using qRT-PCR, including 1L-1β (B), IL-6 (**C**), TNF-α (**D**), and COX-2 (**E**). Results are presented as means ± SD (*n* = 3 independent experiments for (**A**–**E**)); ** *p* < 0.01 indicates a significant difference compared with the untreated control group and ^#^ *p* < 0.05 and ^##^ *p* < 0.01 indicate a significant difference compared with the LPS group. Statistical analyses were performed using a one-way ANOVA.

In addition to NO burst, activated microglia also overexpress other mediators and proinflammatory factors, including ROS, prostaglandins, IL-1β, IL-6, and TNF-α [40]. Our results showed that Pol relieved the ROS production and transcriptional expression of proinflammatory factors, including IL-1β, TNF-α, and IL-6, in LPS-treated BV2 microglia (Figure 3A–D). Additionally, COX-2, a rate-limiting enzyme in the synthesis of prostaglandin E2 (PGE2) that usually shows upregulated expression under the stimulation of inflammation [31], was downregulated by Pol in BV2 microglia treated with LPS (Figure 3E). Collectively, Pol exerted a positive effect on the expression of proinflammatory cytokines and other mediators in BV2 microglia after exposure to LPS.

### 2.4. Pol Attenuated the Activation of NF-κB Pathways in LPS-Treated BV2 Microglia

**Figure 4:**
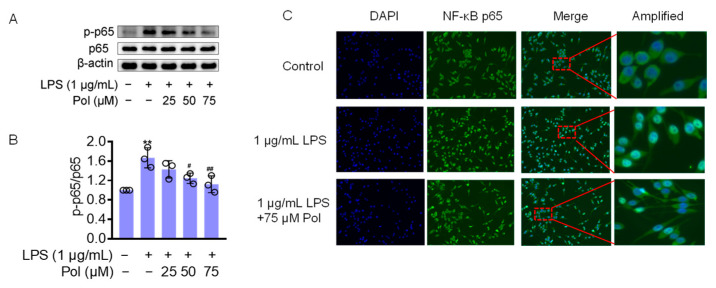
Pol blocked the activation of the NF-κB pathway: (**A**) the protein expression of p-p65/p65 in BV2 microglia exposed to different concentrations of Pol for 1 h prior to the treatment of 1 μg/mL of LPS for another 2 h was detected using a Western blot; (**B**) statistics graph of the protein expression of p-p65/p65; (**C**) the nuclear translocation of NF-κB p65 in BV2 microglia after exposure to indicated concentrations of Pol for 1 h before the treatment of 1 μg/mL of LPS for another 2 h was determined using immunofluorescence. The cells selected by red-dotted boxes in the third column are amplified and shown magnified in the fourth column to their right. Results are presented as means ± SD (*n* = 3 independent experiments for (**A**,**B**)); ** *p* < 0.01 indicates a significant difference compared with the untreated control group and ^#^ *p* < 0.05 and ^##^ *p* < 0.01 indicate a significant difference compared with the LPS group. Statistical analyses were performed using a one-way ANOVA.

The transcription factor NF-κB seems to be a central target for the regulation of neuroinflammation. In LPS-induced microglia, TLR4, the ligand of LPS, triggers IκB kinase (IKK), leading to IκB phosphorylation and the subsequent release of the cytoplasmic NF-κB dimers p50 and p65 from the NF-κB/IκB complex and their translocation to the nucleus [41]. NF-κB is a homo- or heterodimeric transcription factor, which recognizes and binds to p65-responsive elements in the regulatory regions, leading to the transcriptional activation of downstream genes, such as IL-1β, IL-6, TNFα, COX2, and iNOS [42]. They also induce ROS secretion, which is one of the most critical pathways in neuroinflammation-mediated pathology [43,44]. Furthermore, IL-1β and TNF-α have been shown to induce iNOS expression in different cell types by activating NF-κB, thereby initiating an autoregulatory feedback loop [45]. To determine whether Pol influences LPS-induced neuroinflammation through the NF-κB pathway, a Western blot was performed to test the expression level of the above pathway-related proteins. The results showed that the LPS treatment caused a significant increase in phosphorylated NF-κB p65 (p-p65) (Figure 4A,B) and a dramatic increase in NF-κB p65 nuclear translocation (Figure 4C), whereas Pol distinctly attenuated these changes. Together, these findings indicate that the anti-inflammatory effect of Pol is correlated with the suppression of NF-κB pathways.

### 2.5. Asp G at a Low Dose Synergistically Enhanced the NO Inhibition of Pol in LPS-Induced BV2 Microglia

**Figure 5:**
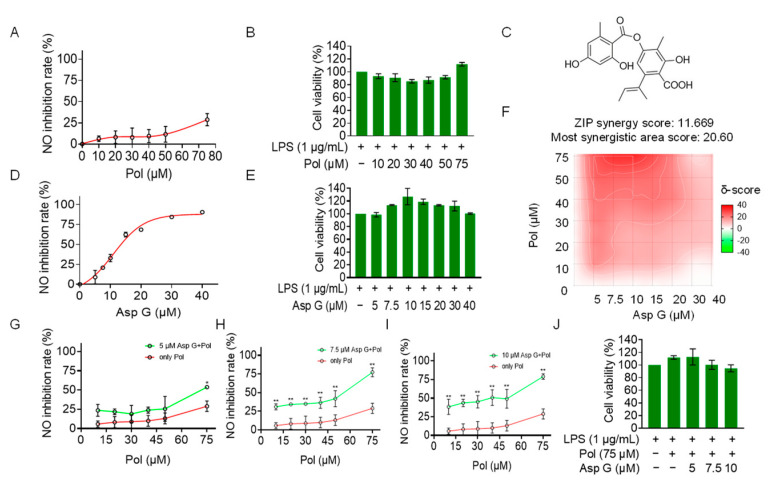
Asp G synergistically increased the NO inhibition of Pol in LPS-induced BV2 cells. BV2 microglia were co-pretreated with the indicated concentration of Asp G and Pol for 1 h followed by stimulation with 1 μg/mL of LPS for another 24 h. Dose–response curve for NO inhibition by Pol (**A**) or Asp G (**D**) in LPS-induced BV2 microglia. The cytotoxicity of Pol (**B**) and Asp G (**E**) in LPS-induced BV2 microglia was tested using CCK8. (**C**) The structure of Asp G. (**F**) Heatmaps of drug combination responses based on NO content using Griess agents. NO inhibition by Pol and 5 μM of Asp G (**G**), 7.5 μM of Asp G (**H**), or 10 μM of Asp G (**I**) together was compared with Pol alone in LPS-treated BV2 microglia. (**J**) The cytotoxicity of LPS-induced BV2 microglia co-exposed to Asp G and Pol was detected using CCK8. Results are presented as means ± SD (*n* = 2 independent experiments); * *p* < 0.05 and ** *p* < 0.01 indicate a statistically significant difference compared to the untreated control group.

Considering that Pol at cell-safe doses does not exert the best anti-neuroinflammatory effect, with 75 μM of Pol inhibiting NO production by less than 50% (Figure 5A,B), a co-administration strategy was used to optimize its activity. We explored whether Pol could exhibit synergistic effects with Asp G in reducing neuroinflammation. Asp G (Figure 5C) itself dose-dependently decreased NO generation in LPS-induced BV2 microglia, with 30 μM of Asp G inhibiting more than 80% of the NO generation (Figure 5D). More importantly, Asp G did not cause cytotoxicity, even at the highest concentration (40 μM) (Figure 5E), presenting a desirable safety level. In the experiment involving the combined inhibition of NO by Asp G and Pol in LPS-induced BV2 microglia, the zero interaction potency (ZIP) synergy score was evaluated to be 11.669, and the most synergistic area score was up to 20.6, indicating there is a synergistic effect between Asp G and Pol (Figure 5F). The concentration ranges of Asp G and Pol were 5–10 μM and 40–75 μM in the region surrounding the highest synergy, respectively, and the drug combination of 7.5 μM Asp G and 75 μM Pol presented an ideal anti-inflammatory effect, with about an 80% NO inhibition rate (Figure 5G–I), without damaging the cell viability (Figure 5J). Collectively, low-dose Asp G synergistically strengthened the NO inhibition of Pol in LPS-induced BV2 microglia.

### 2.6. Asp G Collaborates with Pol to Further Alleviate Neuroinflammation in LPS-Induced BV2 Microglia

**Figure 6:**
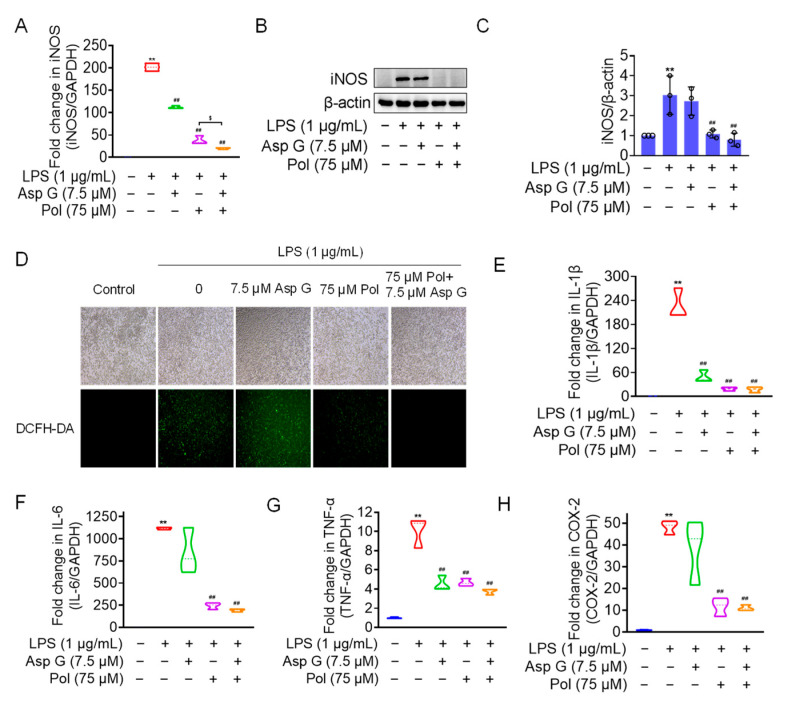
Asp G further attenuated LPS-induced iNOS expression, ROS burst, and related proinflammatory factors in BV2 microglia. BV2 microglia were co-pretreated with the indicated concentration of Asp G and Pol for 1 h prior to the treatment of 1 μg/mL of LPS for another 12 h (**A**,**E**–**H**) or 24 h (**B**–**D**). The transcriptional expression (**A**) and protein expression (**B**,**C**) levels of the iNOS gene when BV2 microglia were co-pretreated with Asp G and Pol prior to the treatment of LPS. (**D**) ROS burst of BV2 microglia treated with Pol, Asp G, and LPS was detected using a DCFH-DA probe under a fluorescence microscope. The transcriptional expression of 1L-1β (**E**), IL-6 (**F**), TNF-α (**G**), and COX-2 (**H**) in BV2 microglia exposed to Pol and Asp G and LPS was detected using qRT-PCR. Results are presented as means ± SD (*n* = 3 independent experiments for (**A**–**C**,**E**–**H**)); ** *p* < 0.01 indicates a statistically significant difference compared to the untreated control group and ^##^ *p* < 0.01 indicates a statistically significant difference compared to the LPS group; ^$^ *p* < 0.05 indicates a statistically significant difference compared to the LPS + Asp G + Pol group. The results were statistically analyzed using a one-way ANOVA.

**Figure 7:**
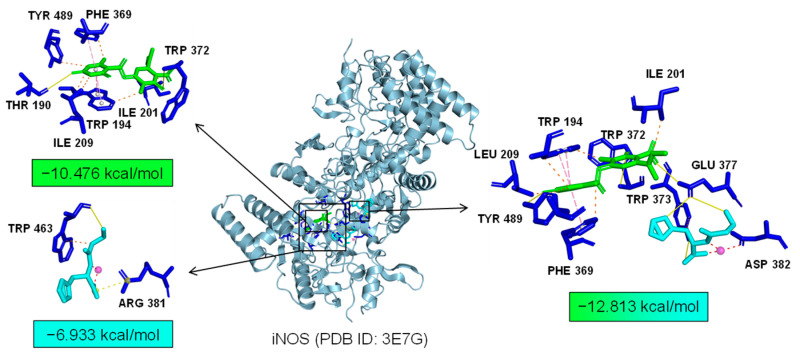
A 3D structure picture of Asp G (green) and Pol (blue) docking separately or together to iNOS. The interaction forces refer to hydrogen bonds (yellow, solid lines), hydrophobic interactions (orange, dotted line), salt bridges (yellow, dotted line), metal–ligand bonds (red, dotted line), and π–π stacking interactions (pink, dotted line).

We the tested the levels of ROS, proinflammatory factors, and inflammatory mediators in LPS-induced microglia under the combined treatment of Asp G and Pol. The results showed that the drug combination further alleviated LPS-induced iNOS expression (Figure 6A–C), ROS generation (Figure 6D), and the expression of proinflammatory factors, including 1L-6 (Figure 6F) and TNF-α (Figure 6G), while there were no changes in the transcriptional expression of 1L-1β (Figure 6E) and COX-2 (Figure 6H). Importantly, the co-treatment of Asp G and Pol mainly led to a significant decrease in iNOS compared to Pol alone in LPS-induced BV2 microglia. This prompted us to consider whether iNOS is a potential target for synergistic anti-neuroinflammation by Asp G and Pol. To evaluate the interaction between the drug combination and iNOS, the binding energy was calculated virtually via molecular docking. As Figure 7 shows, Asp G had potential interactions with 6 residue sites of iNOS via hydrogen bonds, hydrophobic interactions, and π–π stacking interactions, with a binding energy of −10.476 kcal/mol, while Pol interacted with 2 residue sites of iNOS via hydrogen bonds, hydrophobic interactions, and salt bridges, with a binding energy of −6.933 kcal/mol. Surprisingly, when Asp G and Pol co-docked with iNOS, they bound to the 9 amino acid sites of iNOS via hydrogen bonds, hydrophobic interactions, π–π stacking interactions, and metal coordination bonds, with a very low binding energy of −12.813 kcal/mol, suggesting that the co-treatment of Asp G and Pol made the iNOS conformation more stable, which further supported the above inference. Together, the Asp G–Pol combination further attenuated the LPS-induced iNOS expression, ROS burst, and the expression of related proinflammatory factors such as 1L-6 and TNF-α. Moreover, iNOS might be a target for the Asp G–Pol combination to further reduce neuroinflammation in LPS-induced BV2 microglia.

### 2.7. Bioinformatics Analysis of Potential Synergistic Mechanism

**Figure 8:**
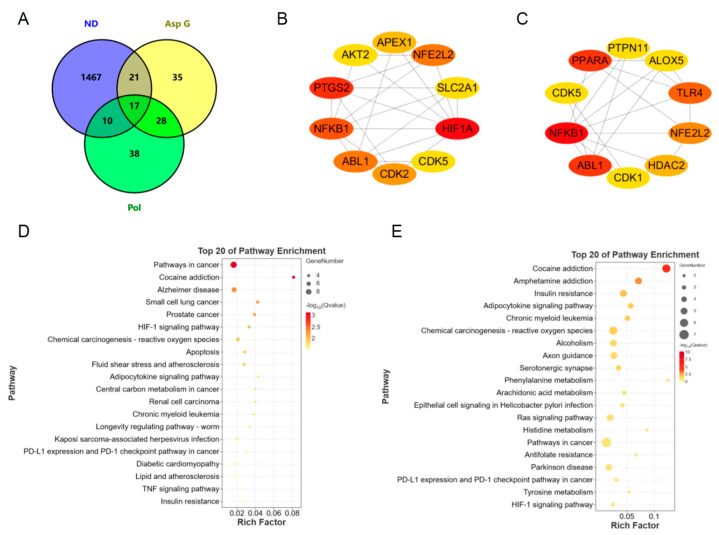
Bioinformatics prediction of synergistic mechanisms between Asp G and Pol against NDs. (**A**) Venny diagram analysis of Asp G- and Pol-related targets using the Super-PRED online website and ND-related targets using the DisGeNET online website. PPI network node diagram of the top 10 core targets of ND-related targets of Pol (**B**) and Asp G (**C**) using Cytoscape. The redder the node color, the better the connectivity of the node. KEGG analysis of Pol-related (**D**) and Asp G-related (**E**) anti-ND targets.

To further explore the possible synergistic mechanism of Asp G and Pol in the treatment of NDs, we predicted and analyzed the ND-related targets of Asp G and Pol using several bioinformatics tools. A total of 93 Pol-related targets and 101 Asp G-related targets were obtained using the Super-PRED website. A total of 1515 ND-related targets were attained from the DisGeNET website. The analysis of the Venny diagram showed that there were 27 overlapping targets between NDs and Pol and 38 overlapping targets between NDs and Asp G (Figure 8A). The top 10 targets of Pol related to NDs are shown in Figure 8B, among which hypoxia-inducible factor 1 alpha (HIF1A), prostaglandin endoperoxide synthase 2 (PTGS2, also known as COX-2), NFκB1, Abelson tyrosine protein kinase 1 (ABL1), and nuclear factor erythroid 2-related factor 2 (NFE2L2, also known as NRF2) were the core targets with high network connectivity. The top 10 targets of Asp G related to NDs are shown in Figure 8C, among which NFκB1, ABL1, peroxisome-proliferator-activated receptor alpha (PPARα), TLR4, and NRF2 were the core targets with high network connectivity. A further Kyoto Encyclopedia of Genes and Genomes (KEGG) analysis of overlapping targets revealed that Pol was mainly involved in cancer pathways, Alzheimer’s disease, cocaine addiction, and the HIF-1 signaling pathway (Figure 8D), and Asp G was mainly involved in cocaine addiction, amphetamine addiction, insulin resistance, and the adipocytokine signaling pathway (Figure 8E). Pol was also involved in the TNF signaling pathway in KEGG enrichment, which was consistent with the result showing that Pol attenuated the activation of the NF-κB signaling pathway stimulated by LPS in BV2 microglia.

**Figure 9:**
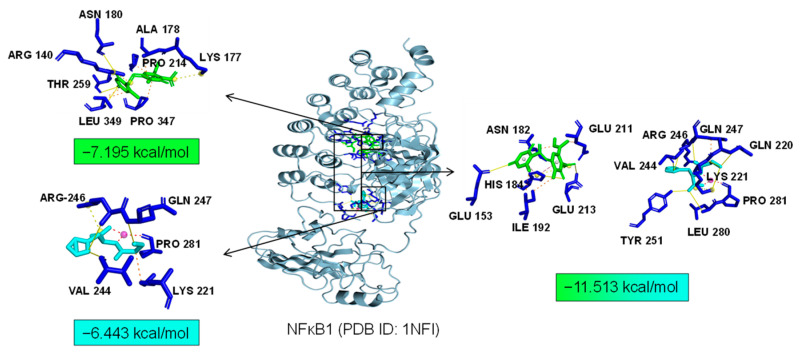
Molecular docking of Asp G and Pol with NFκB1.

**Figure 10:**
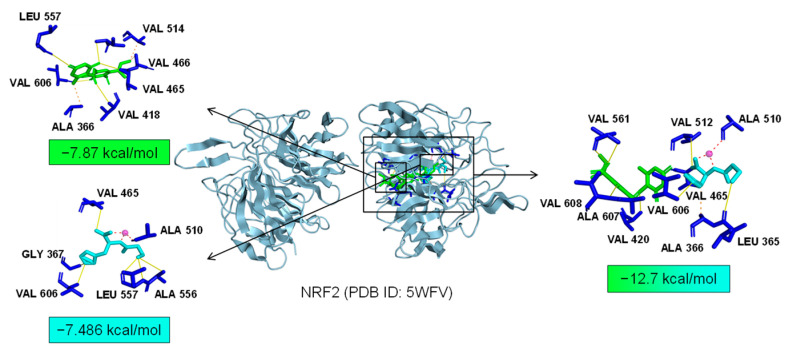
Molecular docking of Asp G and Pol with NRF2.

**Figure 11:**
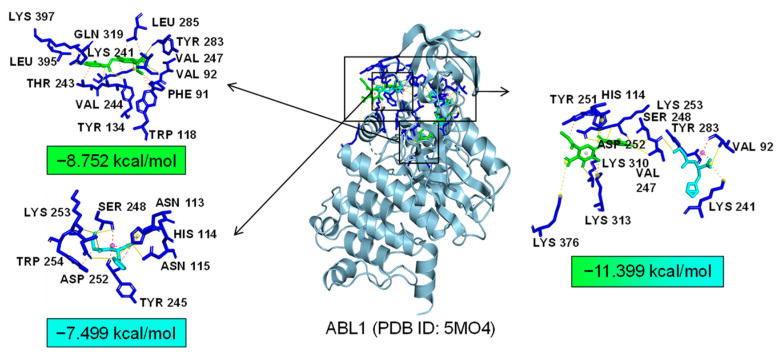
Molecular docking of Asp G and Pol with ABL1.

As common targets of Asp G and Pol, NFκB1, NRF2, and ABL1 were probably involved in their synergistic anti-inflammatory mechanisms. Both NFκB1 and NRF2 are nuclear transcription factors. NFκB1 is one of the components of NF-κB dimer p50/p65, and the dimer translocates to the nucleus after releasing from the NF-κB/IκB complex [41], leading to the transcriptional activation of downstream proinflammatory genes. The analysis of the molecular docking showed that Asp G and Pol were mainly co-docked to the IκBα chain and p65 chain of the NF-κB/IκBα complex, and the co-docking of Asp G and Pol had a stronger interaction, with a binding energy of −11.513 kcal/mol, than either Asp G (−7.195 kcal/mol) or Pol (−6.443 kcal/mol) alone (Figure 9), revealing that the combination of Asp G and Pol might bind to the complex, preventing the release of p50/p65 from the complex and subsequent nuclear translocation. NRF2 regulates the oxidative or xenobiotic stress response and functions as an upstream regulator of cytokine production to repress inflammation [46]. Normally, NRF2 is ubiquitinated by the KEAP1 complex and degraded in the cytoplasm [47]. Our molecular docking result suggested that the co-treatment of Asp G and Pol (−12.7 kcal/mol) formed a more stable combination with the KEAP1–NRF2 complex compared to Asp G (−7.87 kcal/mol) and Pol (−7.486 kcal/mol) alone, and primarily interacted with the KEAP1 chain (Figure 10), which might interfere with the binding of NRF2 to KEAP1, thereby increasing the chances of NRF2’s entry into the nucleus to exert a protective role. ABL1 is a non-receptor tyrosine protein kinase that is involved in a wide range of cellular processes, including cell differentiation, division, and stress responses, and the emerging data support important roles for ABL kinases in pathologies linked to inflammation, such as in NDs and inflammatory pathologies [48]. Typically, the variable site of the ABL1 kinase domain is occupied by its N-terminal myristoyl peptide, which induces cross-linking of the SH3–SH2–kinase structural domain, playing a key role in negatively regulating the activity of the ABL1 kinase and the normal proliferative signaling of the cell. The molecular docking analysis indicated that the co-exposure of Asp G and Pol (−11.399 kcal/mol) gave ABL1 a more stable conformation than either Asp G (−8.752 kcal/mol) or Pol (−7.499 kcal/mol) alone (Figure 11), which might promote or maintain the cross-linking of the SH3–SH2–kinase structural domain.

**Figure 12:**
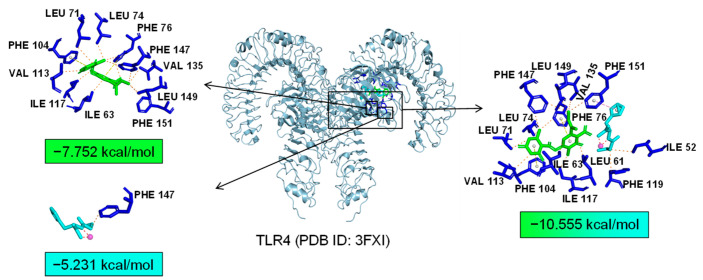
Molecular docking of Asp G and Pol with TLR4.

**Figure 13:**
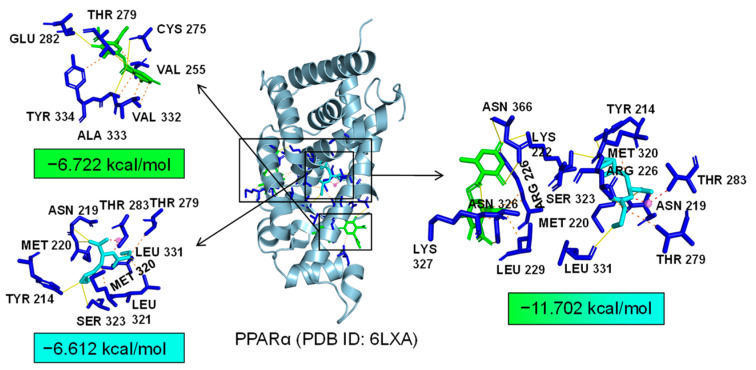
Molecular docking of Asp G and Pol with PPARα.

Additionally, TLR4 and PPARα are unique targets of Asp G. As we know, the activation of the TLR4 receptor triggers a range of inflammatory pathways, so it is possible that Asp G exerts a synergistic anti-inflammatory effect with Pol by inhibiting the activation of this receptor. The results of the molecular docking analysis showed that the addition of Asp G greatly increased the stability of the TLR4 conformation in the position of LPS’s docking pocket, with the binding energy changing from −5.231 kcal/mol to −10.555 kcal/mol (Figure 12). The ability of Asp G and Pol to co-occupy the docking site of LPS in TLR4 may explain their better anti-inflammatory effects. PPARα is a member of the PPAR family that regulates genes involved in glycolipid metabolism and inflammation by targeting PPAR-responsive elements in the promoter regions of target genes [49]. The anti-inflammatory actions of PPARα are thought to be related to its negative interference with the activity of proinflammatory transcription factors, including signal transducer and activator of transcription (STAT), activator protein-1 (AP-1), and NF-κB [50,51,52]. The results of the molecular docking analysis showed that the co-treatment of Asp G and Pol had a lower binding energy of −11.702 kcal/mol compared to either Asp G (−6.612 kcal/mol) or Pol (−6.722 kcal/mol) alone (Figure 13), which indicated that Asp G might promote the activation of PPARα to directly inhibit the binding of NF-κB p50/p65 to the promoters of acute-phase response genes. Asp G might exert a synergistic anti-inflammatory effect through several targets shared or unique to Pol.

## 3. Discussion

There is a well-known saying that the best way to discover a new drug is to start with an old one. The new use of old drugs, as the name suggests, refers to the use of drugs that are already on the market or in clinical trials for new indications, which is an important drug development strategy that not only reduces costs and saves time but also improves the success rate of research and development efforts.

In this study, we have demonstrated the anti-neuroinflammatory properties of Pol, which is traditionally used for gastric ulcers, in LPS-induced BV2 microglia. Our results revealed that Pol significantly decreased the production of NO and ROS while downregulating the expression of inducible iNOS and COX-2. Moreover, it suppressed the expression of key proinflammatory cytokines such as IL-6, TNF-α, and IL-1β. These findings underscore Pol’s potential to mitigate neuroinflammation, a critical mechanism underlying the progression of NDs. Previous studies have suggested that one of the reasons Pol is able to treat gastric ulcers is due to its anti-inflammatory properties in the gastrointestinal tract [53,54,55]. Interestingly, our study found that it also has an inhibitory effect on neuroinflammation in the CNS, and further studies revealed that it might be related to the nuclear translocation of NF-κB and its subsequent transcriptional activation of target genes.

A notable finding of our study is the synergistic effect observed when Asp G was co-administered with Pol in LPS-induced BV2 microglia. Specifically, low doses of Asp G significantly enhanced Pol’s inhibitory effect on NO production, increasing it from approximately 30% to 80%. This synergy suggests that Asp G can potentiate the anti-inflammatory actions of Pol. Our bioinformatics analysis provided further insights into the potential mechanisms underpinning this synergistic Interaction. Several targets, including NFκB1, NRF2, ABL1, TLR4, and PPARα, were identified as possible mediators of the combined effects of Asp G and Pol. NFκB1’s role in regulating the immune response is well established [41], while NRF2 is involved in antioxidant responses [46] and ABL1, TLR4, and PPARα also play significant roles in inflammation and immune modulation [48,49,56]. The synergistic mechanism may be attributed to both overlapping and complementary actions on these targets. Future studies focusing on these targets could elucidate the precise mechanisms through which Asp G enhances Pol’s anti-inflammatory effects.

Despite these promising findings, several limitations of this study necessitate further investigation. Questions remain regarding the pharmacokinetics and bioavailability of Pol, particularly whether it dissociates into L-carnosine and zinc in the gastrointestinal tract and the extent to which it crosses the BBB. The current pharmacokinetics evidence suggest gastrointestinal dissociation [57] but the potential for intact Pol reaching the CNS remains speculative. Detailed multi-organ pharmacokinetic studies are essential to confirm these findings. Even if Pol is not brain-accessible in its intact form, alternative delivery methods such as AAV capsid variants, nanoparticles, or nasal administration could be considered [58,59,60,61,62].

In summary, our study explored the anti-neuroinflammatory potential of Pol and elucidated its underlying mechanisms, primarily through NF-κB pathway inhibition. We also discovered that Asp G can synergistically enhance the anti-inflammatory effects of Pol, suggesting a potent combination therapy. Our bioinformatics analysis highlighted several key targets that could explain the observed synergy, laying the groundwork for future research. These findings provide a strong foundation for potential clinical applications in the treatment of NDs. Further in-depth pharmacokinetic studies and the exploration of delivery mechanisms are essential to fully realize the clinical potential of Pol and its combination with Asp G.

## 4. Materials and Methods

### 4.1. Cell Lines and Culture Condition

The immortalized BV2 murine microglia used in the study were purchased from the China Center for Type Culture Collection (CCTCC) (GDC0311, Wuhan University, China), which was established in 1990 by E. Blasi Nin [63]. The cells were immortalized via the retroviral-mediated transfection of mouse microglia with v-raf/v-myc, retaining a wide range of morphological, phenotypic, and functional characteristics of microglia. They were maintained in Dulbecco’s modified Eagle’s medium (DMEM, C11995500BT, Thermo Fisher, MA, USA) supplemented with 10% (*v*/*v*) fetal bovine serum (FBS, Z7186FBS, Zeta life, USA) and 1% (*v*/*v*) penicillin–streptomycin (15140122, Life Technologies, Carlsbad, CA, USA) in a 37 °C, 5% CO_2_ incubator.

### 4.2. NO Content Measurements

The NO level in the culture medium was evaluated by photometrically quantifying the amounts of stable product nitrite using the NO assay kit (S0021, Beyotime Biotech, Shanghai, China) according to the manufacturer’s methods [64]. Firstly, BV2 microglia with 80~90% confluence were collected and inoculated in 96-well plates at a density of 2 × 10^4^ cells per well for 24 h. Subsequently, the cells were exposed to 1 μg/mL of LPS for 24 h after pretreatment with a series of concentrations of Pol (25, 50, 75 μM) for 1 h. Finally, the absorbance of the cell culture supernatant was measured at 540 nm using a microplate spectrophotometer (Epoch2, Biotek, VT, USA), and the NO concentration was calculated by establishing a standard curve of sodium nitrite. In drug combination assays, the BV2 microglia were pretreated with Pol (10, 20, 30, 40, 50, 75 μM) combined with Asp G (5, 7.5, 10, 15, 20, 30, 40 μM) for 1 h using the checkerboard method before the treatment of 1 μg/mL of LPS for another 24 h. The results were analyzed using the SynergyFinder website (https://synergyfinder.fimm.fi/) accessed on 3 January 2024 [65].

### 4.3. Cell Viability Detection

The cell viability was measured using Cell Counting Kit-8 (CCK-8, S0021, Beyotime Biotech, Shanghai, China) [66]. The BV2 microglia were seeded in 96-well plates at a density of 5 × 10^3^ cells per well for 24 h, and then the cell culture medium was replaced with DMEM containing different concentrations of Pol (25, 50, 75 μM) for another 24 h. Next, 10 μL of CCK-8 reagent was added to each well and the plate was incubated at 37 °C for 1 h. The cell viability was finally estimated by determining the absorbance of each well at 450 nm under a microreader.

### 4.4. ROS Determination

The ROS generation in BV2 microglia was visualized using a 2′,7′-dichlorodihydrofluorescein diacetate (DCFH-DA) (HY-D0940, MCE, NJ, USA) fluorescent probe following the manufacturer’s instructions [67]. The BV2 microglia were seeded in 96-well plates at a density of 2 × 10^4^ cells per well for 24 h, and then the cells were pretreated with a series of concentrations of Pol (25, 50, 75 μM) for 1 h followed by the treatment of 1 μg/mL of LPS for another 24 h. Subsequently, the cells were washed 3 times with DMEM and then incubated with the DCFH-DA probe (10 μM in DMEM) for 0.5 h at 37 °C in the dark. Finally, the cells were washed 3 times with DMEM, followed by a fluorescence determination test using a fluorescent microscope with an FITC channel. In the drug combination assays, the BV2 microglia were pretreated with 75 μM of Pol combined with 7.5 μM of Asp G using the checkerboard method before the treatment of 1 μg/mL of LPS for another 24 h.

### 4.5. Quantitative Real-Time PCR (qRT-PCR)

The total RNA of the BV2 microglia was isolated using TRIzol reagent (AG21101, Accurate Biology, Changsha, China) referring to the classic method [68]. Briefly, the cells were seeded in 6-well plates at a concentration of 6 × 10^5^ cells per well for 24 h, following which they were treated with the indicated drugs (Pol, Asp G, or LPS) at the indicated times. The RNA purity and concentration were detected using an ultraviolet-visible spectrophotometer (DS-11, DeNovix, Wilmington, MA, USA), and subsequently the RNA samples that met the requirements were reverse-transcribed into cDNA using HiScript III RT SuperMix for qPCR (+gDNA wiper) (R323, Vazyme, Nanjing, China) following the manufacturer’s instructions. The RT-PCR was performed using ChamQ Universal SYBR qPCR Master Mix (Q711, Vazyme, China) through the RT-PCR detection system (CFX96 Touch, Bio-Rad, Hercules, CA, USA). The relative expression of genes was calculated using the 2-ΔΔCt method, and GAPDH was chosen as the reference gene. The sequences of primers used in this study were as follows: GAPDH (mouse: sense, 5′-AGGTCGGTGTGAACGGATTTG-3′, antisense, 5′-TGTAGACCATGTAGTTGAGGTCA-3′), cyclooxygenase-2 (COX-2) (mouse: sense, 5′-TTCAACACACTCTATCACTGGC-3′, antisense, 5′-AGAAGCGTTTGCGGTACTCAT-3′), IL-6 (mouse: sense, 5′-TAGTCCTTCCTACCCCAATTTCC-3′, antisense, 5′-TTGGTCCTTAGCCACTCCTTC-3′), IL-1β (mouse: sense, 5′-GCAACTGTTCCTGAACTCAACT-3′, antisense, 5′-ATCTTTTGGGGTCCGTCAACT-3′), TNF-α (mouse: sense, 5′-CCCTCACACTCAGATCATCTTCT-3′, antisense, 5′-GCTACGACGTGGGCTACAG-3′), iNOS (mouse: sense, 5′-GTTCTCAGCCCAACAATACAAGA-3′, antisense, 5′-GTGGACGGGTCGATGTCAC-3′), MMP9 (mouse: sense, 5′-CTGGACAGCCAGACACTAAAG-3′, antisense, 5′-CTCGCGGCAAGTCTTCAGAG-3′).

### 4.6. Western Blotting

The BV2 microglia were seeded in 6-well plates at a concentration of 6 × 10^5^ cells per well for 24 h and then treated with Asp G or LPS at the indicated times. Next, the cells were cracked in ice-cold RIPA lysis buffer (P0013B, Beyotime Biotechnology, China) containing 1 mM of PMSF (ST506, Beyotime Biotechnology, China) or 1 × protease and phosphatase inhibitor cocktail (P1050, Beyotime Biotechnology, China) to obtain the total protein sample. The Western blot assay was performed in reference to Liao’s method [69]. Briefly, equivalent denatured protein samples were separated using SDS-PAGE, then these separated proteins in gels were transferred to nitrocellulose filter (NC) membranes (10600001, Amersham™, Cytiva, DE, USA). Subsequently, the NC membranes were blocked in 1 × PBST (tris buffered saline with 0.1% (*v*/*v*) tween-20) with 5% (*w*/*v*) skim milk powder or 5% BSA for 2 h at room temperature, then incubated with specific primary antibodies overnight at 4 °C and secondary antibodies for 2 h at room temperature. Finally, immunoreactive bands of proteins were indicated using a hypersensitive ECL chemiluminescent reagent (F03, Willget Biotechnology, Hangzhou, China). The following antibodies were used: mouse anti-β-actin lgG (sc-47778, Santa Cruz Biotechnology, Santa Cruz, CA, USA), mouse anti-nos2 lgG (sc-7271, Santa Cruz Biotechnology, USA), mouse anti-p-p65 lgG (sc-136548, Santa Cruz Biotechnology, USA), mouse anti-p65 lgG (sc-8008, Santa Cruz Biotechnology, USA), horseradish peroxidase (HRP)-coupled horse anti-mouse lgG (7076, Cell Signaling Technology, Danvers, MA, USA), DyLight 488-coupled goat anti-mouse IgG (A23210, Abbkine, Santa Ana, CA, USA). The gray value of each immunoblot band was calculated using ImageJ 1.53q (National Institutes of Health, Bethesda, MD, USA).

### 4.7. Immunofluorescence

The immunofluorescence experiment was performed to detect the nuclear translocation of p65 [70]. Briefly, the BV2 microglia were seeded onto poly-L-lysine-coated (5 μg/mL in PBS) slips in 48-well plates at a concentration of 3 × 10^4^/well for 24 h, following which they were treated with Pol and LPS at the indicated times. Next, the cells were rinsed with PBS three times each time for 3 min and then fixed with a 4% paraformaldehyde fixing solution (P0099, Beyotime, Shanghai, China) for 20 min. Subsequently, the cells were washed with PBS again and permeabilized with enhanced immunostaining permeabilization buffer (P0097, Beyotime, China). Next, the cells were blocked with 1% (*w*/*v*) BSA (in PBS) for 1 h after washing and then directly incubated with the primary antibody (in 1% (*w*/*v*) BSA) at 4 °C overnight. After washing, the cells were incubated with the goat anti-mouse IgG secondary antibody DyLight 488 at 37 °C for 1 h in the dark. The nuclei were counterstained with DAPI (1 μg/mL) (C1002, Beyotime Biotechnology, Shanghai, China) after washing. The cellular localization of the protein was observed under a fluorescence microscope with the same exposure settings as for the comparison group.

### 4.8. Network Pharmacology Prediction

The network pharmacology analysis was performed to predict the anti-PD effects of Asp G and Pol [71]. The human targets of Asp G and Pol were collected from the Super-PRED database (https://prediction.charite.de/) [72] accessed on 18 May 2024 by submitting the SMILES code or PubChem-name. Meanwhile, the disease targets were obtained from Disgenet (https://www.disgenet.org/) accessed on 18 May 2024 using “neurodegenerative disorder” as the search term. Next, their overlapping targets were analyzed and exported using Venny 2.1 (https://bioinfogp.cnb.csic.es/tools/venny/) accessed on 18 May 2024. Subsequently, Gene Ontology (GO) and KEGG enrichment analyses were performed using the R package clusterProfiler [73]. Additionally, protein–protein interaction (PPI) networks of overlapping targets were analyzed using the STRING database (https://cn.string-db.org/) accessed on 31 May 2024, and the core targets were identified based on the number of nodes connected by a node and visualized using Cytoscape_v3.9.1.

### 4.9. Molecular Docking

The potential interactions of small and large molecules were evaluated via semi-flexible molecular docking using AutoDock Vina 1.2.3. The large-molecule files (.pdb) were downloaded from the PDB database (https://www.rcsb.org/) accessed on 11–13 July 2024. The small-molecule files (.mol2) were drawn using ChemOffice2020. Firstly, the system’s coordinate files (.qdbqt) of large molecules were prepared by removing redundant ligands, H_2_O, or unnecessary proteins and adding hydrogens. The files (.pdbqt) of small molecules were obtained after setting active key numbers. The next step was to set the range of ligand search sites by adjusting the size of the “grid box” according to the site of the original ligands or the predicted docking pocket. Next, “autodock vina” was run using the config file achieved in the previous step. Then, the achieved autodock vina result (.pdbqt) was saved as the file (.pdb), then the ligand and receptor files were integrated and further visually analyzed using the Protein–Ligand Interaction Profiler online website (https://plip-tool.biotec.tu-dresden.de/plip-web/plip) [74] accessed on 13–15 July 2024 and Pymol 2.1.0. When two ligands were simultaneously docked to a receptor, additional information about other ligands was added to the config file before running.

### 4.10. Statistics Analysis

The statistical analysis was performed using GraphPad Prism 8. A one-way ANOVA was used to analyze the significance of differences between groups. Here, *p*-values < 0.05 were considered statistically significant.

## Figures and Tables

**Table 1:** Drug-likeness properties.

Property	Value	Potential for Development as a Neurological Drug
Lipinski rule	Accept (MW: 289.03, logP: −2.875)	Excellent
Pfizer rule	Accept (logP: −2.875, TPSA: 123.17)	Excellent
Golden Triangle rule	Accept (MW: 289.03, log D: −1.848)	Excellent
GSK rule	Accept (MW: 289.03, logP: −2.875)	Excellent
Blood–brain barrier penetration	0.825	Excellent
Human intestinal absorption	0.506	Medium

Note: Lipinski rule: MW ≤ 500, logP ≤ 5, Hacc ≤ 10, Hdon ≤ 5. If two properties are out of range, poor absorption or permeability is possible, while one is acceptable. Pfizer rule: Compounds with a high log P (>3) and low TPSA (<75) are likely to be toxic. Golden triangle: 200 ≤ MW ≤ 50, −2 ≤ logD ≤ 5. Compounds satisfying the golden triangle rule may have a more favorable ADMET profile. GSK rule: MW ≤ 400, logP ≤ 4. Compounds satisfying the GSK rule may have a more favorable ADMET profile. Blood–brain barrier penetration: values range from 0 to 1, and the closer the value is to 1, the easier the compound crosses the blood–brain barrier. Scale: 0–0.3: poor; 0.3–0.7: medium; 0.7–1.0: excellent. Human intestinal absorption: values range from 0 to 1, whereby the closer the value is to 1, the more difficult the compound is to be absorbed by the intestines. Scale: 0–0.3: excellent; 0.3–0.7: medium; 0.7–1.0: poor.

## Data Availability

The data presented in this study are available upon request from the corresponding author.

## Supplementary Materials

The following supporting information can be downloaded at https://www.mdpi.com/article/10.3390/md22070324/s1: Table S1. ADMET properties of polaprezinc.
