# Supplementary material for: Aspergillusidone G Potentiates the Anti-Inflammatory Effects of Polaprezinc in LPS-Induced BV2 Microglia: A Bioinformatics and Experimental Study
Source: Mar Drugs. 2024 Jul 19;22(7):324. doi: 10.3390/md22070324 (PMC11278036; doi:10.3390/md22070324)
Supplement: Supplementary file 1 [file marinedrugs-22-00324-s001.zip › marinedrugs-3102447-supplementary.pdf]

Table S1 ADMET properties of Polaprezinc

Property	Value	Decision
**sorption**		
Caco-2 Permeability	-5.946	Poor
MDCK Permeability	2.2e-05	Excellent
Pgp-inhibitor	0.002	Excellent
Pgp-substrate	0.819	Poor
Human Intestinal Absorption (HIA)	0.506	Intermediate
20% Bioavailability ($F_{20\%}$)	0.008	Excellent
30% Bioavailability ($F_{30\%}$)	0.032	Excellent
**Distribution**		
Plasma Protein Binding (PPB)	9.797%	Excellent
Volume Distribution(VD)	0.559	Excellent
Blood-Brain Barrier (BBB) Penetration	0.825	Poor
The fraction unbound in plasms (Fu)	89.78%	Excellent
**Metabolism**		
CYP1A2 inhibitor	0.003	-
CYP1A2 substrate	0.358	-
CYP2C19 inhibitor	0.022	-
CYP2C19 substrate	0.051	-
CYP2C9 inhibitor	0.012	-
CYP2C9 substrate	0.92	-
CYP2D6 inhibitor	0.008	-
CYP2D6 substrate	0.062	-
CYP3A4 inhibitor	0.044	-
CYP3A4 substrate	0.041	-
**Excretion**		
Clearance (CL)	2.219	Poor
$T_{1/2}$	0.874	-

**Toxicity**		
hERG Blockers	0.034	Excellent
Human Hepatotoxicity (H-HT)	0.063	Excellent
Drug Induced Liver Injury (DILI)	0.085	Excellent
AMES Toxicity	0.018	Excellent
Rat Oral Acute Toxicity	0.089	Excellent
Maximum Recommended Daily Dose (FDAMDD)	0.069	Excellent
Skin Sensitization	0.829	Poor
Carcinogenicity	0.047	Excellent
Eye Corrosion	0.004	Excellent
Eye Irritation	0.064	Excellent
Respiratory Toxicity	0.049	Excellent
